# Fluctuations in serum sodium level are associated with an increased risk of death in surgical ICU patients

**DOI:** 10.1186/cc10752

**Published:** 2012-03-20

**Authors:** Y Sakr, S Rother, AM Ferreira, C Ewald, P Dünich, K Reinhart

**Affiliations:** 1 Friedrich Schiller University Hospital, Jena, Germany

## Introduction

Dysnatremia may have an impact on outcomes in critically ill patients, but this has not been widely investigated in surgical ICU patients. We investigated the epidemiology of dysnatremia in a large cohort of surgical ICU patients and evaluated the possible influence of the time of acquisition of dysnatremia and fluctuations in serum sodium concentrations on hospital mortality in these patients.

## Methods

All patients admitted to the ICU between January 2004 and January 2009 were included retrospectively in this study. Hyponatremia was defined as a serum sodium concentration (sNa) <135 mmol/l and hypernatremia as a sNa >145 mmol/l. Hyponatremia was defined as a sNa less than 135 mmol/l and hypernatremia as a sNa greater than 145 mmol/l. Patients were classified according to the onset of dysnatremia into those who had abnormal sodium concentrations in the initial blood sample, analyzed within 2 hours of admission to the ICU, or those acquiring dysnatremia thereafter. We performed a logistic regression multivariate analysis with hospital outcome as the dependent factor to investigate the possible influence of dysnatremia on hospital outcome.

## Results

Of the 10,923 surgical ICU patients included in the study, 1,215 (11.2%) had hyponatremia and 277 (2.5%) hypernatremia at admission to the ICU. Among patients with normonatremia at admission to the ICU (*n *= 9,431), the incidence of ICU-acquired dysnatremia was 31.3%. Dysnatremia present at ICU admission (OR = 2.53; 95% CI: 2.06 to 3.12, *P *< 0.001) and ICU-acquired dysnatremia (OR = 2.06; 95% CI: 1.71 to 2.48, *P *< 0.001) were independently associated with an increased risk of in-hospital death. Dysnatremia at ICU admission (OR = 1.23; 95% CI: 1.01 to 1.50) was associated with a higher risk of in-hospital death, compared to ICU-acquired dysnatremia. Fluctuation in serum sodium concentration was also independently associated with an increased risk of in-hospital mortality; both in patients who remained normonatremic (>6 mmol/l/ICU stay) and those with dysnatremia (>12 mmol/l/24 hours or >12 mmol/l/ICU stay).

## Conclusion

Dysnatremia was common in surgical ICU patients and was independently associated with an increased risk of in-hospital death in these patients. Dysnatremia at ICU admission was associated with a higher risk of death compared to ICU-acquired dysnatremia. Fluctuations in serum sodium concentrations were independently associated with an increased risk of in-hospital death, even in patients who remained normonatremic during the ICU stay.

